# You prime what you code: The fAIM model of priming of pop-out

**DOI:** 10.1371/journal.pone.0187556

**Published:** 2017-11-22

**Authors:** Wouter Kruijne, Martijn Meeter

**Affiliations:** Department of Experimental and Applied Psychology, Faculty of Behavioural and Movement Sciences, Vrije Universiteit, Amsterdam, The Netherlands; Centre de neuroscience cognitive, FRANCE

## Abstract

Our visual brain makes use of recent experience to interact with the visual world, and efficiently select relevant information. This is exemplified by speeded search when target- and distractor features repeat across trials versus when they switch, a phenomenon referred to as intertrial priming. Here, we present fAIM, a computational model that demonstrates how priming can be explained by a simple feature-weighting mechanism integrated into an established model of bottom-up vision. In fAIM, such modulations in feature gains are widespread and not just restricted to one or a few features. Consequentially, priming effects result from the overall tuning of visual features to the task at hand. Such tuning allows the model to reproduce priming for different types of stimuli, including for typical stimulus dimensions such as ‘color’ and for less obvious dimensions such as ‘spikiness’ of shapes. Moreover, the model explains some puzzling findings from the literature: it shows how priming can be found for target-distractor stimulus relations rather than for their absolute stimulus values per se, without an explicit representation of relations. Similarly, it simulates effects that have been taken to reflect a modulation of priming by an observers’ goals—without any representation of goals in the model. We conclude that priming is best considered as a consequence of a general adaptation of the brain to visual input, and not as a peculiarity of visual search.

## Introduction

Because our human visual system has a limited capacity, it constantly selects only a subset of input at the expense of other information in a visual scene. This occurs by means of *visual attention* [1, for a review]. Ideally, attention would always select the information that is currently most relevant to us, but doing this is not straightforward. For example, physically conspicuous stimuli tend to capture attention, even when we already know them to be irrelevant [2]. Moreover, in many situations we will not even know beforehand what aspects of visual information will be relevant.

Because of this, selecting what we selected before can be a useful heuristic. Take for example a hot night where one may want to hunt down mosquitoes in the bedroom. Mosquitoes can be hard to spot, but as soon as the first one has been found, it often becomes easier to find others: our visual system has swiftly recalibrated to find again what has just been found. Experiments on visual attention and selection have illustrated that the visual system is indeed strongly modulated by past experience, both on short- [3–5] and longer time scales [6–9].

One phenomenon that clearly demonstrates the effects of recent experience is intertrial priming of pop-out [5]. In typical priming experiments, participants are presented with a search display where a target stimulus has one of two colors (classically red and green), and a set of distractor stimuli of the other color. Participants do not know in advance what the color of the target will be, but regardless, this is an easy task. Because the target ‘pops out’ from the rest of the items it can be found through a ‘bottom-up’ search, based on local contrasts in visual input [1, 10, 11]. Nevertheless, performance on this task is strongly affected by the recent past: search is hampered when the colors of the target and distractor switch from trial to trial, and is facilitated when they stay the same. It thus seems that already early on, low-level processing of a visual scene is affected by recent visual experience (see also [12, 13]).

Many studies have concluded that such intertrial priming automatically changes the ‘weights’ of different visual features in the task [5, 14–17]. This claim gives rise to several questions. Most prominently, *what* is actually being weighted in feature weighting? And how does this affect future attentional deployments?

An intuitive answer would be that priming modulates the responsiveness or baseline activity of visual neurons coding for observed target- and distractor feature values. Recent findings on priming of pop-out, however, suggest that it may not be so straightforward. Several studies supported the idea that perhaps not the value of targets and distractors is primed in visual search, but rather their relation. One illustrative experiment [18] involved a singleton search where stimulus colors are either yellow, orange or red. It was found that repetition priming not only occurred for straight repetitions of target-distractor stimuli, but also for displays with different stimuli where the relation between target and distractors remained constant. So for example: an orange target among yellow distractors would be primed by a preceding red target among orange distractors, because the target remained the ‘reddest’ item in the display. Note that in this case, priming was found even though the preceding distractor color (orange) became the new target color. Conversely, a repeated orange target among yellow distractors would be harder to find after an ‘orange target-red distractor’ trial, even though target color remained constant. In this case, the relation reversed: the target was the yellowest element in the display, and became the reddest.

Such *relational priming* has been observed across various dimensions: colors of different palettes, size, luminance [18–20], and even higher-order shape features such as ‘spikiness’ of star-shaped stimuli, and the ‘complexity’ of visual shapes [21]. These results suggest that intertrial priming does not act upon the features in the scene per se, but rather on a representation of the relation between the target and the distractors. However, no proposal has been made for what such a representation would look like or how it would arise.

Another puzzling finding is that priming may depend on the task that is to be performed on the next trial. In one pop-out visual search experiment [22] search displays always contained two separate singleton items: a color singleton (red versus green) and a shape singleton (circle versus rectangle). Before each trial, a cue would instruct the participant whether to search for (and respond to) the color singleton or the shape singleton. It was found that repetition of the singleton only affected search for the dimension that was relevant to the current goals, resulting in *goal-dependent priming*. Remarkably, this effect was independent of what had been the task on the previous trial. It seemed as if priming of either color or shape was associated with its dimension. If that dimension then turned out to be irrelevant, priming would no longer affect the subsequent search.

Both relational- and goal-dependent priming seem to contradict the simple conceptualization of priming as the automatic weighting of target- and distractor features. However, because there is very little clarity on what ‘feature weighting’ actually is, it is hard to determine whether these findings truly warrant a different explanation of priming. For the case of relational priming, it has indeed been argued that the finding is consistent with a theory of absolute feature weighting [20]. Similarly, the extent to which goal-directed effects on visual attention should be attributed to automatic, (goal-*independent*) priming is strongly debated [23, 24]. However, we feel these debates will remain unresolved and vulnerable to much ambiguity until we construct a more detailed hypothesis of how priming effects arise in the brain.

To bring theoretical clarity, we set out to provide the first comprehensive, explicit, computational account of intertrial priming. We aimed to develop a model that is as simple and as general as possible, and that is consistent with current views on visual search.

These considerations led us to use the AIM model (Attention by Information Maximization [25, 26]) of bottom-up visual processing as a basis. AIM is a salience model, devised to describe how certain locations in a visual scene come to be more conspicuous than others. Although AIM is primarily derived from computational principles, its computations are highly consistent with the organization of the human visual system. Crucially, the model does not rely on a hand-crafted feature base (in contrast to most salience models [27–29], see [30] for a review). Instead, it learns a sparse visual representation from the statistical regularities in visual input. This approach ensured that we did not unconsciously rely on custom-built representations that had been chosen or modified to explain the priming phenomena of interest.

We present fAIM (feature-weighted AIM), which is essentially AIM extended with a single computational step in which the ‘gain’ of visual feature channels can be modulated. For pop-out visual search, we propose that this modulation can arise from only two processes: accommodation to recent visual experience, and an up-regulation of those features associated with detecting the target stimulus. Here, we show that this minimal approach to priming can account for a surprisingly wide range of effects, including classical priming effects and their time course, relational priming in various dimensions, and task-dependent priming.

## Analysis

### The AIM model of bottom-up visual processing

The AIM model consists of two distinct phases: (1) the computation of sparse feature base to represent visual information, and the computation of salience maps. Here, we will give a conceptual outline of both. For more implementation detail, we refer to the Supporting Information S1 Text, or the original publications on AIM [25, 26].

#### Visual features

**Fig 1:**
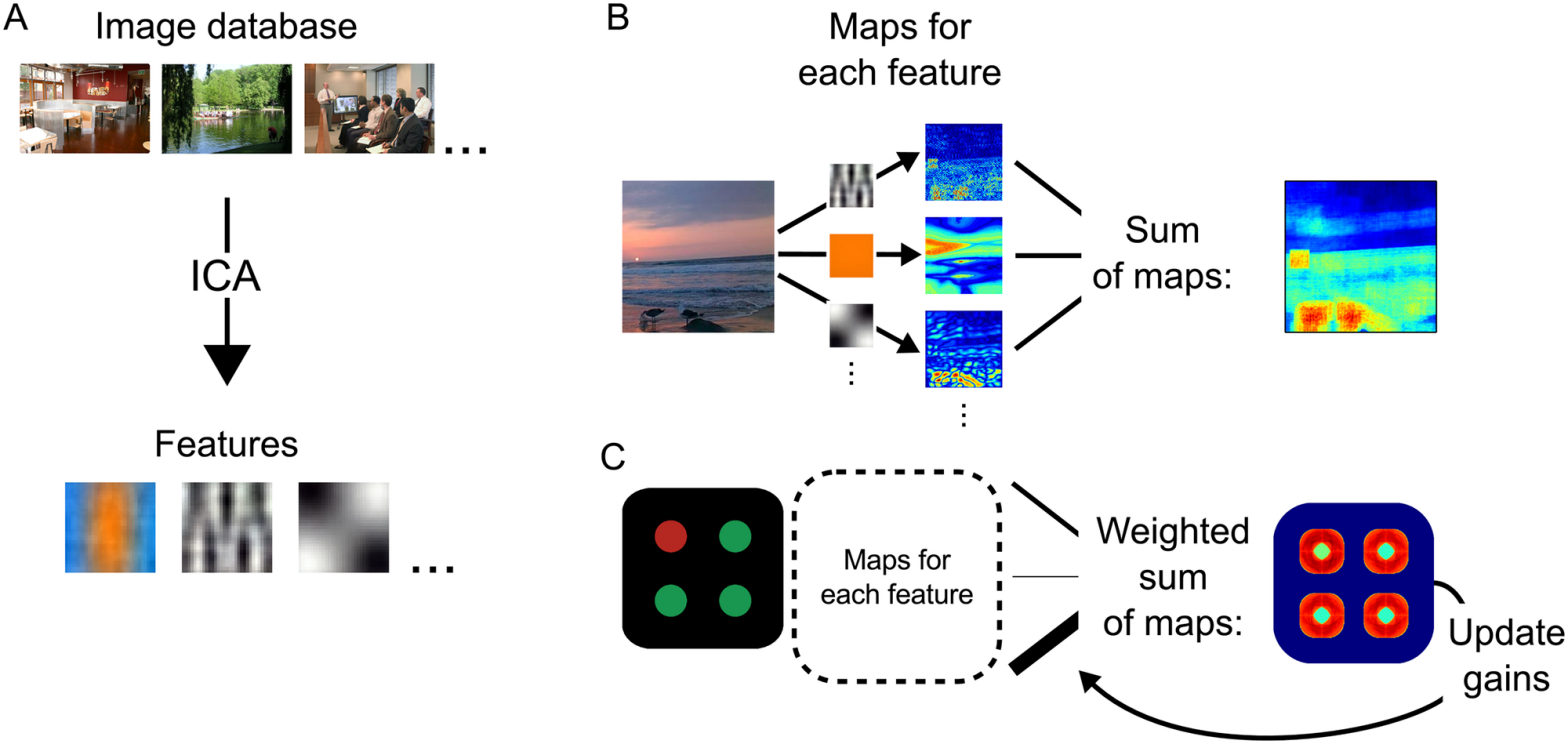
**A** The features that are used in AIM and fAIM are derived from the statistics of visual input. By means of an independent component analysis (ICA) performed on a large image database, features are obtained that have response properties similar to those of neurons in low-level visual areas. **B** In AIM, the response of each of these features is used to generate one ‘salience map’ for each feature channel, based on the principle of self-information. The sum of these maps across all channels yields the overall salience at each location. **C** fAIM additionally assigns a gain to each feature channel that weights the feature’s contribution to the overall salience. The channel gains are adjusted on every trial, as a function of target- and distractor salience values within a channel.

The feature basis used in AIM represents a sparse code for visual information. This representation is derived from the natural statistics of typical visual input. To this end, a large number of small image patches is extracted from a collection of natural color photographs (we have used the SUN Database [31]). Then, Independent Component Analysis is performed on these patches, to derive a sparse spatiochromatic basis that define the features (Fig 1A). All simulations in this study utilized the same component analysis, which yielded 54 features that explained at least 95% of the variance in visual input. These components make up the visual features that are used in the salience computation.

Interestingly, these features have response profiles that resemble those of neurons in early visual areas, suggesting that our visual system indeed implements such a sparse visual representation. (See [32–35] for a more extensive argument for such a sparse visual code in the brain, and see [36–38] for other successful applications using similarly derived visual representations.)

#### Self-information and visual pop-out

AIM defines salience as the ‘uniqueness’ of a location in the visual scene with respect to its surround. From an information theoretic viewpoint, such uniqueness is measured by the amount of self-information [39], which is a function of how likely the visual input at that location is. AIM computes the likelihood at a location by constructing image histograms for all the feature values surrounding that location. From these histograms the model estimates the joint probability of the particular combination of feature values found at a location.

In the simplest implementation of AIM, the ‘surround’ is defined by the entire image. This way, the feature histograms only need to be constructed once, which can be used to determine self-information at each location. This is an oversimplification, as it renders the model insensitive to local variations in feature frequency. Effects of local contrast can be incorporated into AIM by computing different feature histograms at every location, based on the local surround. However, this comes at a large computational cost and has been shown to yield only small improvement of performance [25]. Therefore, we will here also adopt the simpler implementation.

AIM can take any input image and produces a salience map that marks conspicuous locations. As is common for salience map models, its performance has been validated by comparing these maps to fixation data of human observers [40]. The present work aims to simulate human performance in singleton search, indexed by response times (RTs) and the amount of fixations on the target. This requires a single measure of pop-out that can be used to compare the model to empirical data. We derive this measure from the salience maps as the extent to which the average salience value of the target differs from the average salience value of all stimuli in the image.

### The fAIM model

In AIM, each feature channel contributes equally, and total salience is determined as the sum of self-information in each feature channel (Fig 1B). fAIM generalizes this model and assigns a dynamic weight or ‘gain’ *g*_*f*_ to each feature channel (Fig 1B). These gains are equal at initialization, and are modulated throughout sequences of search trials.

We assume that two distinct processes affect the gains of different feature channels to functionally inhibit or excite them on a subsequent trial. Evidence for a dissociation between excitation and inhibition can be observed behaviorally in the individual variation across observers [15]. We relate the down- and up-regulation of gains in singleton search to two distinct processes: First, features that have previously yielded high salience values will have a discounted gain on the subsequent trial. This reflects the fact that repeated presentation of a visual stimulus will reduce its salience [25, 29], which is characterized by attenuated responses in the brain [3, 41, 42]. Second, features that evoked high salience in the *target* will have a positive gain change, which may reflect a strengthening of synapses triggered by the inherent reward in finding a target. The resulting intertrial gain modulation for each feature channel *f* in our model is defined as:

$$\begin{array}{c} \Delta g_{f}=w_{e}{\bar{S}}_{f}^{T}-w_{i}{\bar{S}}_{f}^{A} \end{array}$$

where ${\bar{S}}_{f}^{T}$ and ${\bar{S}}_{f}^{A}$ refer to the average salience value within channel *f* for the ‘Target-’ and of ‘All’ stimuli respectively. The parameters *w*_*e*_, *w*_*i*_ scale the relative contribution of positive (excitatory) and negative (inhibitory) gain modulations: they function as positive and negative learning rates, respectively. Although they are separated in this equation to highlight how they likely reflect different processes, *w*_*e*_ = *w*_*i*_ = 1 in all simulations that follow. As such, they reflect a single scaling parameter of the magnitude of priming effects. Of note, none of the other parameters in the model have any qualitative influence on the results, which rely on changes in stimulus salience, expressed in arbitrary units.

Note that the computation of gain modulations is equivalent to our definition of stimulus pop-out: both rely on the salience contrast between the target and distractors. Priming will therefore increase the gain of those feature channels that contribute to this contrast, thereby increasing target pop-out in a repeated display. One interpretation of this is that priming thus regulates the gains of feature channels in correspondence with the signal-to-noise ratio within these channels [43, 44].

It would be implausible to assume that effective gain changes in the brain would occur linearly and unbounded. This would, after sufficient repetition, render the brain wholly unresponsive to certain visual input. We therefore constrained the gains in our model between a lower bound of 0, and an upper bound of twice their starting value. These limits were enforced via a sigmoid function, which rendered gain changes incrementally smaller as they approached these bounds.

### Comparing fAIM to intertrial priming experiments

We compared model performance in simulations of several key experiments of priming in visual search. Images resembling the visual displays from these experiments were used as input, and the model produced a measure of pop-out *P*, in the manner discussed above. More detail on the stimulus displays is given as supporting information S2 Text.

Typically in these experiments the difference in performance on repetition and switch trials is taken as a measure of priming. The model implementation allows us to isolate the priming effect of different trial combinations by comparing target pop-out to baseline pop-out *without* priming, i.e. with equal gains for all feature channels. Priming effects can be quantified in the model as the change in target pop-out (labeled Δ*P*) evoked by different trial combinations.

The comparison of these model priming effects to the experiments will be qualitative. For each experimental condition, fAIM produces a deterministic value that reflects the change in salience due to priming (Δ*P*). With empirical data, experimenters have to rely on indirect measures of pop-out (e.g. RT, the amount of first fixations on a target item, or response accuracy). It is beyond the scope of the present work to assess how the model priming measure quantitatively relates to RT distributions or error percentages, but we assume that Δ*P* monotonically maps onto average priming effects found in experiments, be it indexed by RT or as a proportion of fixations. As such, the model should be able to account for the presence or absence of priming in a given condition, and to some extent for the size of effects in different conditions within one experiment.

## Results

### Feature priming of pop-out with two stimuli

Our first simulations assess fAIM in a ‘standard’ priming of pop-out paradigm, with two possible stimuli. Each input image contained either a green target circle among red distractor circles, or vice versa.

**Fig 2:**
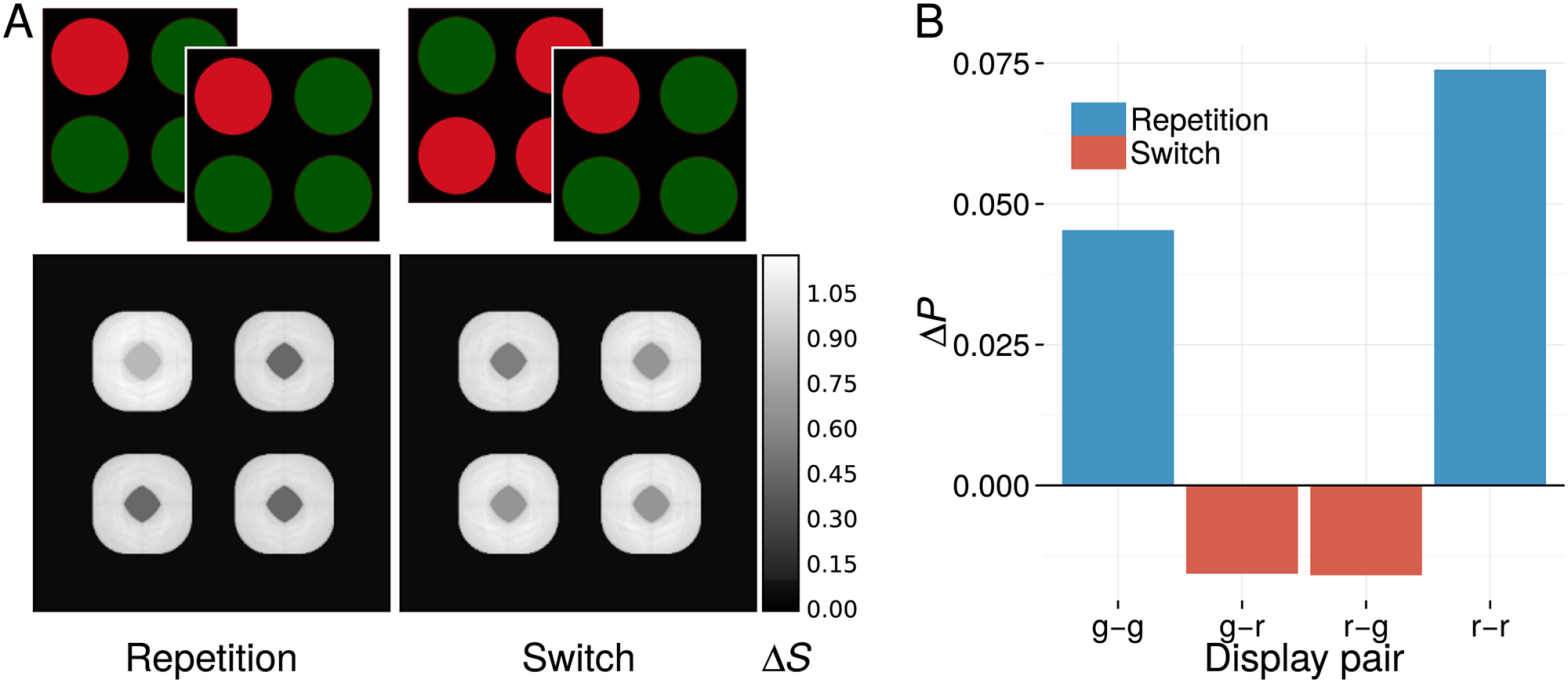
**A** The change in salience Δ*S* of the target (top left stimulus) and distractors, averaged across repetition and switch trial pairs. Priming enhances the salience of both targets and distractors relative to baseline, but for repetition trials this increment is higher for targets than for distractors. After a switch, their difference has decreased. **B** As a result, priming only enhances target pop-out for those trial combinations that constitute a repetition. The effects of repetition or switches for opposite trial combinations are not necessarily identical in magnitude. In this and all subsequent figures, red colors are used to either indicate a switch trial, a decrease in gain, or a decrease in target-pop-out whereas the color blue is used for repetitions and increases in pop-out.

Fig 2A illustrates the effect of both repetition- and switch conditions on the output salience values of the four different stimuli (Δ*S*). The repetition effect is visible in this figure as increased salience (lighter hue) for the upper left target in the case of a repetition relative to a switch. It is clear from this figure that priming more strongly affects the salience of the edges of the stimuli than the center. This is because the majority of the features are variable types of edge detectors, and changes in their gain will thus have less effect on the center of the stimulus. It may seem surprising that the change in salience is positive at both the target and the distractor locations in both conditions, and that priming thus enhances the salience of both in fAIM. The reason for this is that since the target is a unique singleton, it will likely ‘pop-out’ to some extent in most feature channels. As a result, the *gain change* will be positive for most feature channels, resulting in an increase in overall salience. However, critical to the ease of the search (or: the amount of *pop-out* *P*), this change in salience differs for the target and the distractors. The net result is a larger difference in salience between the two when the target repeats, and a smaller difference when the target switches.

Fig 2B illustrates this net pop-out change Δ*P* for the four possible color combinations. This figure also illustrates that the amount of priming is not necessarily symmetric between two colors. Here, increases and decreases in pop-out are somewhat more pronounced for trial pairs that had a red target on the preceding trial. This is the consequence of an asymmetry in the baseline red-green and green-red salience in AIM. Interestingly, we have have observed such a color asymmetry ourselves in several psychophysical experiments, although they are rarely of primary interest and tend to vary across participants. Often, RTs are found to be faster for red targets than for green targets.

**Fig 3:**
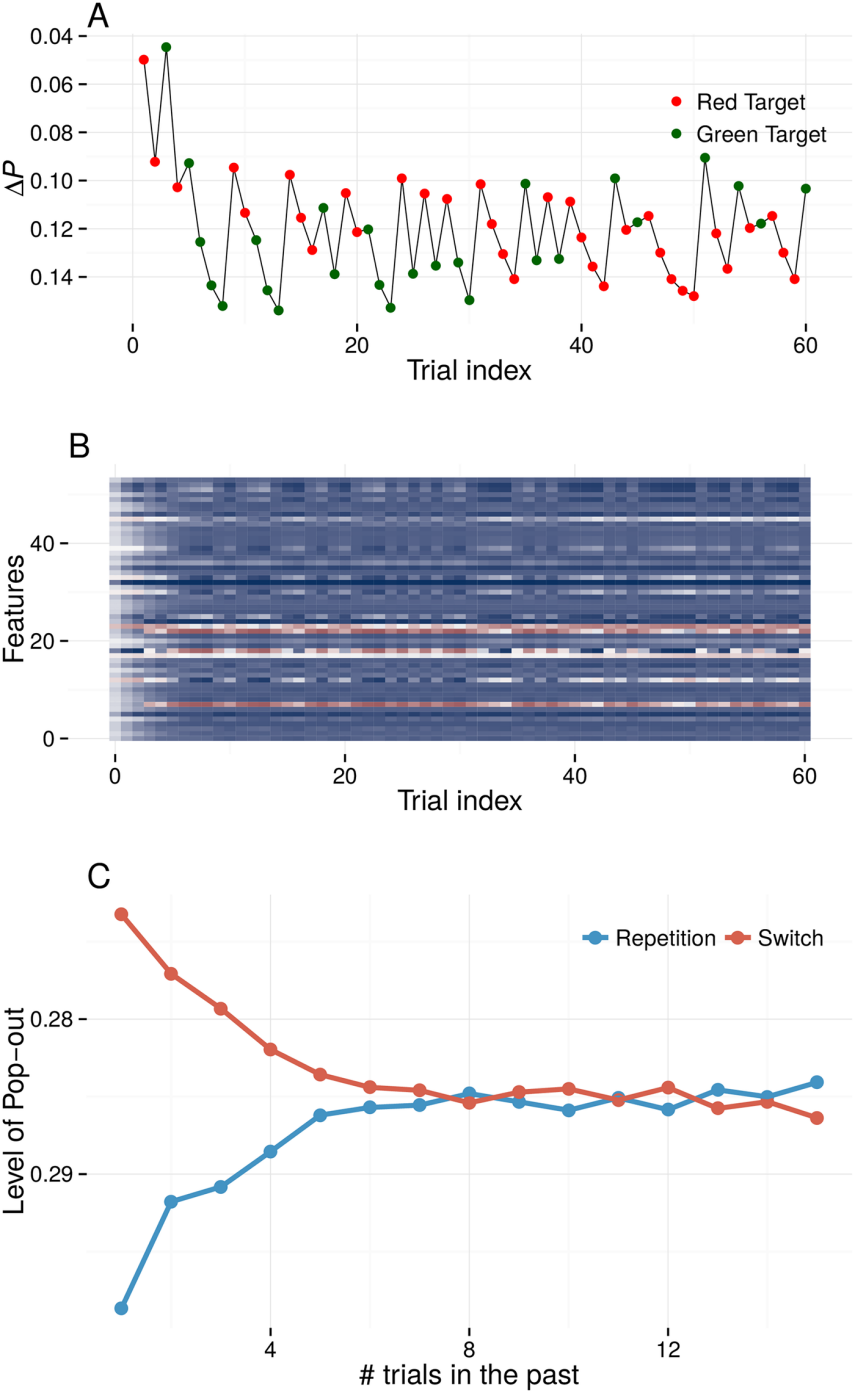
**A** Priming, measured as the change in target pop-out Δ*P*, throughout a trial sequence simulated with fAIM. The scale has been inverted because increases in Δ*P* should yield decreases in RT, the common empirical measure. At the start of a sequence, both repetition and switch trials tend to increase target pop-out compared to baseline. Later in the sequence, the fluctuations in priming across repetition- and switch-trials match those in typical experiments [45]. **B** The gain change relative to baseline for all feature channels. Increases are depicted as blue shading, decreases in red, and the baseline value in white. Most gains increase, but the magnitude and direction of these changes varies across features. The observed priming effects result from this concert of gain modulations. **C** Target pop-out as a function of whether the target repeats or switches compared to *k* trials ago (scale again inverted). Simulated priming decays over 5–8 trials, as is commonly found in the literature.

Note that the intertrial ‘distractor enhancement’ that is shown in Fig 2A only occurs for the first few trials in a sequence. Fig 3A illustrates priming throughout a random sequence of 60 trials, relative to the amount of pop-out in the baseline salience map. The first trials show an overall increase in pop-out for both repetition and switch-trials, i.e. nonspecific facilitation of the search. This is not unlike the first trials in an experiment, where observers quickly adapt to the task parameters (although such startup effects probably also reflect learning processes other than priming—such as general task acquisition and response preparation). Once the gains are in a range that is appropriate for the task, repetitions and switches strengthen and decrease the ease of the search, respectively, in a manner that is commonly observed during a pop-out visual search. Once the weights are in a stable regimen, the pattern of repetition and switch-trials resembles that of RTs in typical priming of pop-out experiments [45].

The evolution of gains for the sequence shown in Fig 3A is depicted in Fig 3B. In this figure, blue shading indicates an increase of the gains relative to baseline, and red indicates a decrease. As was explained above, the gains predominantly increase, although some gains become smaller. Importantly, this figure also highlights that in fAIM, priming is not determined by one, or a few features alone. Rather, the majority of visual features will modulate their gains in response to the visual input, which taken together gives rise to priming effects.

The sequential effects that arise in such a trial sequence are summarized in Fig 3c, which depicts the amount of pop-out *P* given that the target is either a repetition or switch compared to the target *k* trials in the past (following [5]). As has often been found, priming decays over some 5-8 trials [5, 16, 17].

In all following simulations, we will only measure priming as the change in target pop-out Δ*P* relative to the baseline level, as shown in Fig 2B.

### Relational priming in various stimulus dimensions

**Fig 4:**
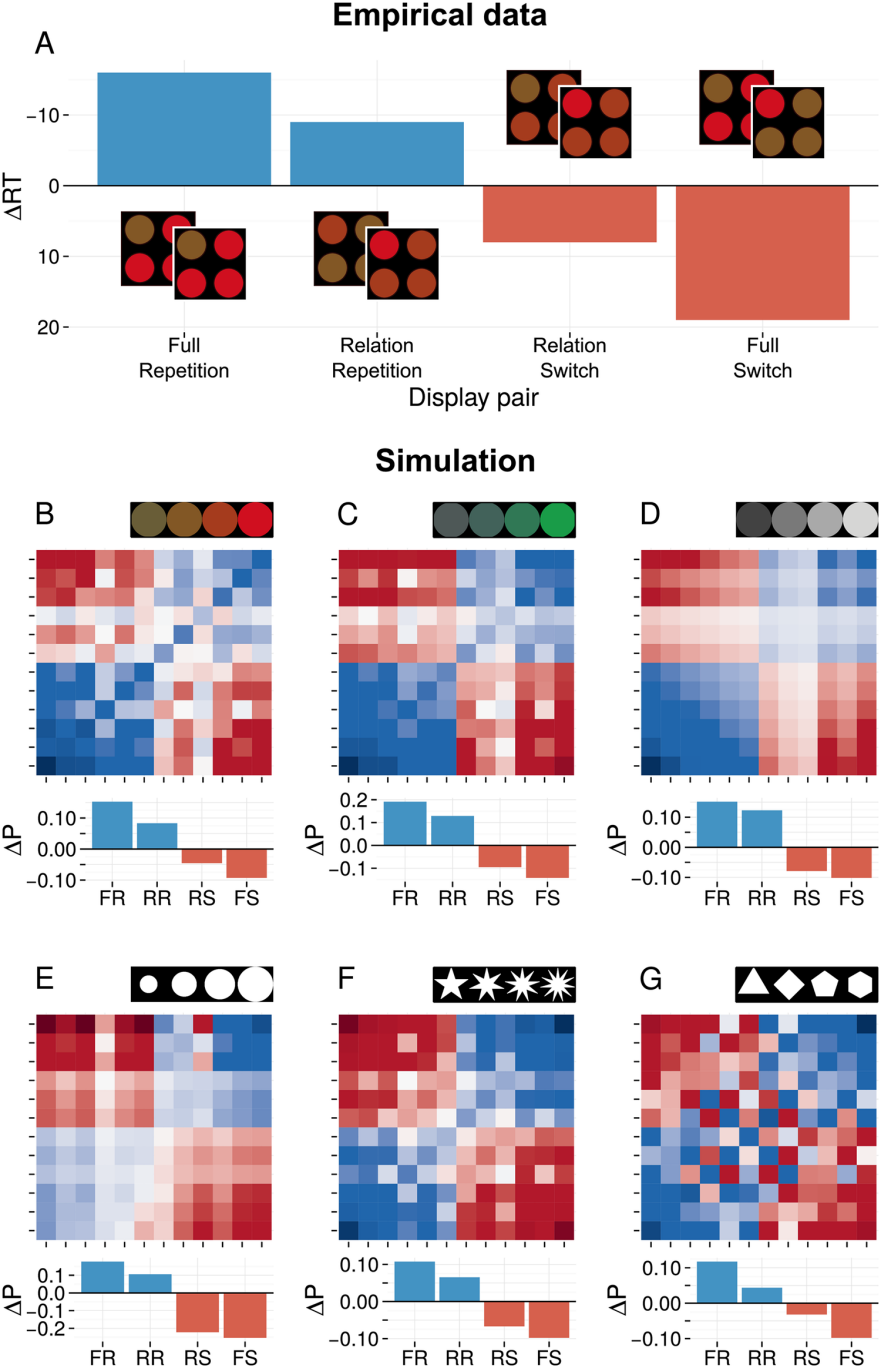
**A** Typical data for relational priming measured as the change in RT (in ms) compared to the average across all conditions (y-axis inversed). Full repetitions (FR) and switches (FS) of target-and distractor roles yield repetition benefits and switch costs, respectively. Similar priming is observed for combinations where the target-distractor relation repeats (RR) or switches (RS). Data from [20]. **B-G** Simulation results for all possible trial combinations in six different visual feature dimensions. Dark blue and dark red sections of the grid indicate trial combinations with strong priming and switch costs, respectively; light shading indicates minimal priming effects. The x- and y-axes mark all possible display combinations for trial *n* − 1 and *n*, respectively. Axes are sorted based on the target-distractor feature distance. As a result, intertrial relations are clustered: RR in the lower left and upper right quadrant, RS in the upper left and lower right quadrants, and FR and FS on the diagonals. The bar graphs summarize the priming effects in these conditions. All dimensions yield relational priming with increased pop-out when the relation remains identical, but decreases when the relation reverses.

Fig 4A illustrates empirical data [20] for the relational priming effect that was described in the introduction: when stimulus values vary from yellow to red, then only trial pairs where the target-distractor relation reverses yield switch costs, and pairs where the relation remains the same yield priming benefits. To investigate this effect, we defined six different stimulus dimensions, modeled after stimuli that have been used in experiments to elicit relational priming [18, 20, 21]: two color gradients (blue to green and yellow to red); luminance (darker to brighter); size (smaller to larger); and two more abstract shape dimensions: stars with an increasing number of spikes; and regular polygons with an increasing number of edges. We define four stimulus values along each dimension, and create a display for each possible target-distractor combination (4 × 3 = 12 displays). Each display contained one target stimulus, and three distractor stimuli. We compute the intertrial priming effect for each of the 12 × 12 = 144 possible intertrial combinations.

Fig 4B–4G depict the priming effects produced by the model for every possible trial combination in each simulated stimulus dimension. For these figures, displays were sorted based on the ‘feature distance’ between target- and distractor values. The x-axis corresponds to the display index on trial *n* − 1, and the y-axis to the index on trial *n*. The colors of the tiles mark the resulting change in pop-out, with blue shading marking an increase in pop-out, and red shading a decrease. Note that because of the way the x-and y-axes are sorted, the bottom-left and top-right quadrants group trial combinations with a relation repetition (RR), and the top-left and bottom right those combinations where the relation switches (RS); The tiles on the diagonals correspond to Full Repetitions (FR) and full switches (FS) of targets and distractors. Each plot is accompanied by a bar chart summarizing relational priming as the average pop-out change in FR trials, RR trials (excluding FR), RS trials (excluding FS), and FS trials.

The figures show that fAIM gives rise to relational priming: The overall change in pop-out is clearly different for RR and RS trials. Increases and decreases in pop-out, marked by blue and red shading, are clustered and coarsely confined to RR and RS trials, respectively. For some dimensions clustering is particularly clear, such as for the blue-green color dimension or the luminance dimensions. The accompanying bar chart shows there is approximately equal priming for FR and RR trials, and equal switch costs for FS and RS trials. In other dimensions, such as for the polygons, positive and negative priming effects are somewhat less well confined to stimulus relations. From their bar charts it can be observed that priming is larger for full repetitions and switches than for merely relational repetitions and switches. This has similarly been observed empirically [20].

Taken together, these simulations demonstrate that relational priming effects arise in fAIM, a model that does not use an explicit relational code.

### Relational priming of complex shapes

In the preceding simulations, relational effects were less consistent for the polygon search dimension than for the other stimuli. There are several mismatches between our simulations and the exact experiment that could underly this. For example, the orientation of the stimuli was always the same in our simulation, whereas stimuli were randomly rotated in the experiment [21]. We therefore ran additional simulations that more closely corresponded to the design of the original experiments on relational priming in shapes.

In the original experiment, relational priming was studied using blocks with two types of search displays. Each display type was characterized by a specific predefined Target-Distractor value combination. Display types would repeat or switch unpredictably. Every display contained one target and seven distractors, evenly distributed on a circle, and each stimulus in a display had a random orientation. Performance was quantified by the proportion of trials where the first eye movement landed on the target singleton. Relational priming was assessed in a block, measured as performance differences on trials where the display type repeated or switched.

The precise display types (i.e. target-distractor combinations) that were used in different blocks are summarized in Table 1. The numbers in this table correspond to either the number of spikes on the stars or the number of sides on the polygons. Asterisks mark intertrial combinations where the target value remained constant. In each pair, the first digit refers to the value of the targets, the second of the distractors. The original study on relative shape priming also reports from an experiment with stimuli of increasing ‘complexity’; however, complexity increases in this study coincided with increases in stimulus size, a confound that was also acknowledged. Since our simulations already showed strong relational effects of size, so we omitted that experiment from our simulations. All stimuli used in these simulations have an identical surface area.

**Table 1:** Target–distractor combinations for relational priming with shapes.

Condition	Label	Stars (A)	Stars (B)	Polygons
Full Switch	FS	5–7, 7–5	7–9, 9–7	
Relation Switch	RS	5–7, 9–7		3–6, 4–3
Relation Repeat	RR	5–9, 7–9	5–7, 7–9	3–4, 4–8
Relation Switch (constant target)	RS*		7–5, 7–9	5–3, 5–10
Relation Repeat (constant target)	RR*			3–5, 3–10

For our simulations we generated fifteen trials for each condition in each block, with randomly rotated stimuli. Priming was computed for all possible trial combinations, excluding the repetition of identical displays (as display repetitions with identically rotated stimuli would not have occurred in the original experiment). The resulting priming values were averaged across trial combinations with display type repetitions, compared to switches.

**Fig 5:**
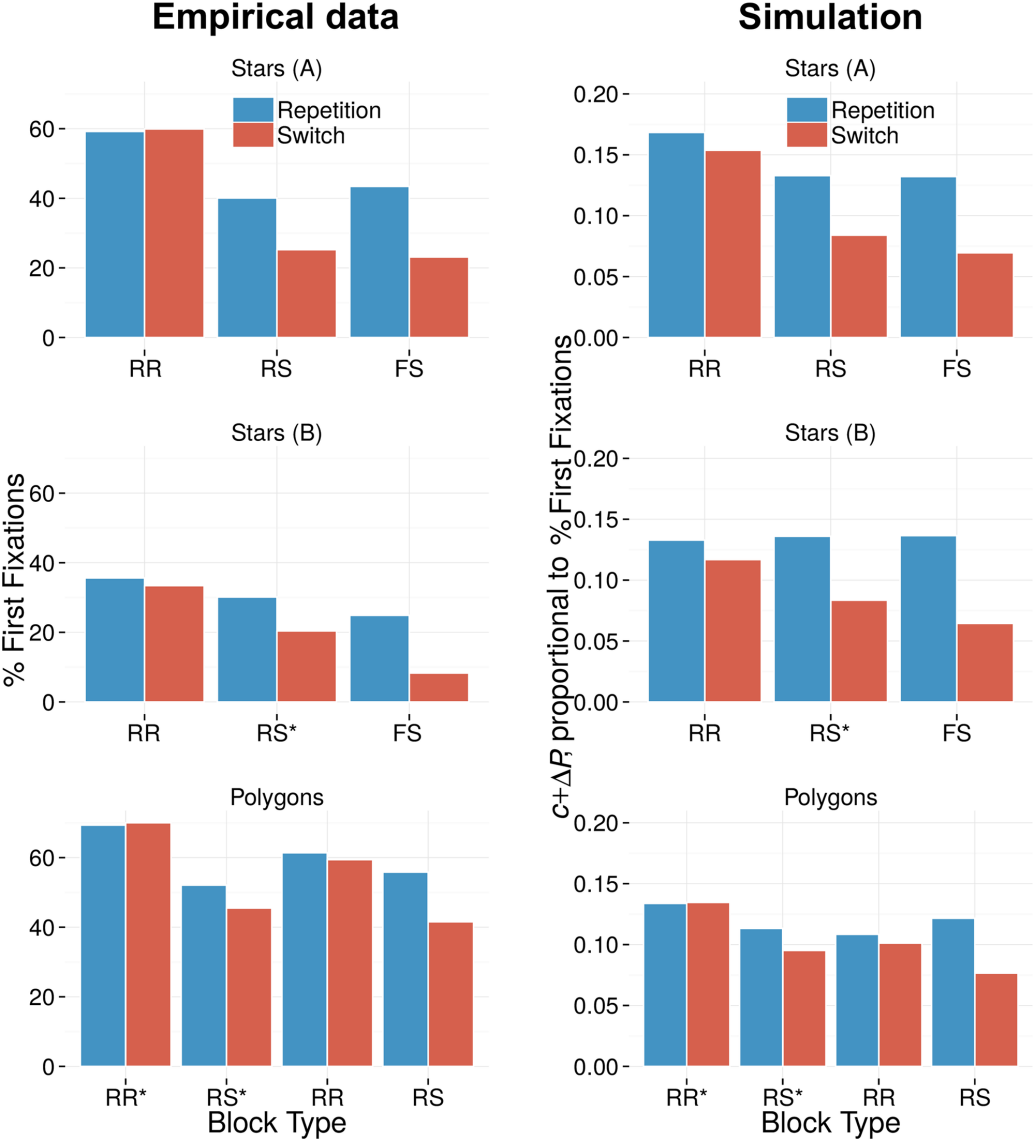
Relational priming of ‘higher level’ shape representations is found both empirically (left) and with fAIM (right). Stimuli are described in Table 1. In the simulations, priming is quantified by the change in pop-out (Δ*P*), and a single constant has been added to these scores. The resulting values are proportional to the measure used in the empirical data: the mean percentage of first fixations on the target, reported in [21]. Critically, priming of display type is only observed in blocks where switches result in a relational switch (with repetition of the target stimulus, RS* or without, RS) or a full switch (FS), and not for blocks where the relation only repeats (RR* and RR).

The results for the three simulated experiments are summarized in Fig 5 (left), and the mean empirical data are depicted for comparison right. To facilitate the comparison, a constant baseline (*c* = .1) was added to all simulation results, to yield visually similar graphs. In the empirical data, the repetition of a display type was statistically different from switches only in those blocks where a switch constituted a relation switch (RS) or a full switch (FS). For those blocks where the different display types had the same relation (RR), no significant difference was found. For fAIM, these effects can be inspected by considering the difference in the amount of priming exerted by display type repetitions versus switches. As is is clear in these figures, this difference is strongly attenuated or completely absent in those blocks where the stimulus relation remains the same across display types.

To a large extent, the model also captures the relative magnitude of the priming effects. For example, in both star experiments, the relational priming effect is greater in the FS-condition than in the RS-condition. For the polygon experiment, the priming effect is smaller in the relation-switch-block where the target remained the same throughout (RS*) than where it did not (RS).

### Relative and absolute attentional capture

The simulations presented above illustrate that relational priming effects are produced by fAIM, a model that implements priming through gain changes in its feature base. This implementation seems to argue for an absolute feature weighting account, rather than a relational code for feature guidance. Because of this contrast, it is of interest that relational attention effects have not only been reported in the context of intertrial priming. In a set of studies investigating attentional capture with consistent target- and distractor features, it was shown that an additional singleton can capture attention in similar, relational fashion [46]. Interestingly, one experiment additionally showed that such relative attentional capture could be modulated, and that attention could be ‘trained’ to be driven by absolute rather than relative feature values [47]. In the present section, we explore these effects to investigate whether relative capture might also arise in the visual representation put forward by fAIM.

**Fig 6:**
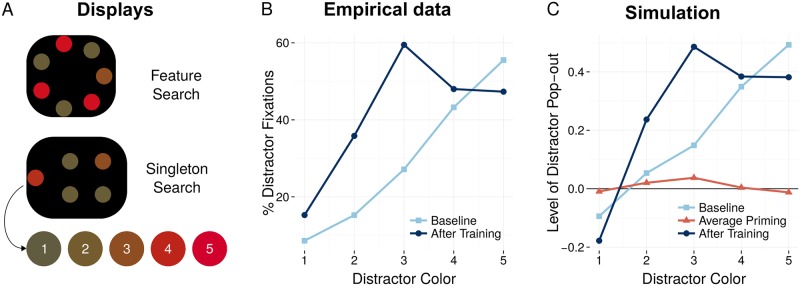
Relative and absolute attentional capture in the additional singleton paradigm. **A** The displays used in the experiment [47] and simulations. In both feature- and singleton search, the target is always orange. During feature search, the target is embedded among more red and more yellow non-targets. During singleton search, the non-targets are always yellow, and an additional singleton distractor is added with a color value anywhere from yellow to red (disks labeled 1–5). **B** Participants who first engage in singleton search show relative capture: fixations on the distractor almost linearly increase as it becomes more red (Baseline, light blue curve). Participants who first ‘train’ in feature search show absolute capture in subsequent singleton search, where the eyes are drawn most to an orange distractor (After Training, dark blue curve). **C** Simulations with fAIM reveal that with homogeneous feature weights, salience of the distractor in singleton search corresponds to relative capture (Baseline, light blue curve). The average priming effect elicited in a feature search block boosts the salience of orange stimuli, and attenuates the salience of yellow and red items (Average priming, red curve). If a scaled version of this priming effect is added to the baseline curve, it gives rise to absolute, rather than relative capture (After Training, light blue curve).

To this end, we simulated this experiment [47, Expt 3] (Fig 6A). In the singleton search condition of that experiment, participants consistently searched for an orange target among three yellow distractors, each randomly positioned on one of four fixed locations. Either on the left or on the right of these stimuli, an additional distractor item was presented that was never the target and had one of five colors that ranged from yellow to red. Of interest was the amount of oculomotor capture by this distractor, which was assessed as the percentage of first fixations that went to this distractor item. Capture was found to be relative: the eyes were captured more often by distractors that were more red compared to those that were more yellow (summarized in Fig 6B as ‘Baseline’, light blue curve). Note that this was found even though the target was consistently orange, the redder distractors would capture the eyes more often than an orange distractor. The experiment additionally contained blocks with a reversed relation, where the target was orange among red, which yielded conceptually identical results. The same held for our simulations of this condition, and these results are therefore not reported here.

To explore these results with fAIM we computed a salience map for singleton search displays like those in the experiment, and computed the amount of pop-out of the additional distractor item for each of its five possible color values. The result is plotted as ‘Baseline’ in Fig 6C (light blue curve). The level of pop-out increases approximately linearly across stimulus values ranging from yellow to red, which is very similar to the experimental data pattern. What this suggests is that relative capture in singleton search does not necessarily reflect relative feature guidance: AIM (and thus fAIM) predicts a similar pattern of relational pop-out, based purely on the physical salience within the display.

Critically, a subset of participants in the experiment engaged in a feature search task in the first half of the experiment, before performing the singleton search. During feature search the target was also orange, but it was on each display accompanied by different distractors that could be both more yellow and more red. Strikingly, these observers, who first engaged in feature search, showed a somewhat different pattern of capture during the subsequent singleton search task. Capture was a combination of relative and absolute: red items still captured the eyes more often than yellow items did, but orange items captured the eyes even more often than red items did (Fig 6B, ‘After training’). This effect was robust, as it did not seem to attenuate over the course of the singleton search trials.

Such long-term effects are not modeled by fAIM in its current form: the priming effects it models will dissipate within 5–8 trials (Fig 3C). Nevertheless, we show here that the model can offer insight into the nature of such guidance, as it is closely related to the gain modulations in intertrial priming.

We let fAIM process a sequence of 25 feature search trials, and modulate the gains in the same way as before. After every trial, we independently assessed how these gain modulations affect the pop-out of the additional distractors in the display used in singleton search. This yielded a ‘priming effect’ of the feature search on each possible distractor value. The average of these priming effects is depicted in Fig 6C as ‘Average priming’ (red curve). The curve shows that priming exerted by feature search trials increases pop-out for an orange distractor, and attenuates pop-out of red and yellow distractors. If this effect is scaled (multiplied by a constant, 8.0), and then added to the baseline salience of these stimuli in the singleton display, the result is very similar to the empirically observed amount of capture across stimuli (compare Fig 6B and 6C, dark blue curves labeled: ‘After Training’).

This simulation illustrates that the long-lasting absolute guidance that was found after feature-search could reflect a stronger, more pronounced expression of the same mechanisms that govern intertrial priming. An obvious difference, however, is their time course. It seems that feature search can give rise to long-lasting modulations of attention to particular features, whereas priming in singleton search is only short-lived (see also [6, 7, 47–49]). This point is addressed further in the Discussion.

### Is priming modulated by the relevance of the dimension?

Priming in fAIM is established by weighting feature channels, dependent on their contribution in dissociating the target from the distractors. Priming is therefore considered a by-product of target selection, which will affect subsequent visual processing, regardless of the upcoming task. This view is at odds with the goal-dependent priming hypothesis [22]. This hypothesis was based on an empirical observation that the effects of target repetitions or switches in a dimension that becomes task-irrelevant are smaller than what would be expected if priming results from absolute feature weighting. Here, we re-examine the experiment that gave rise to the goal-dependent priming hypothesis by simulating it with fAIM.

**Fig 7:**
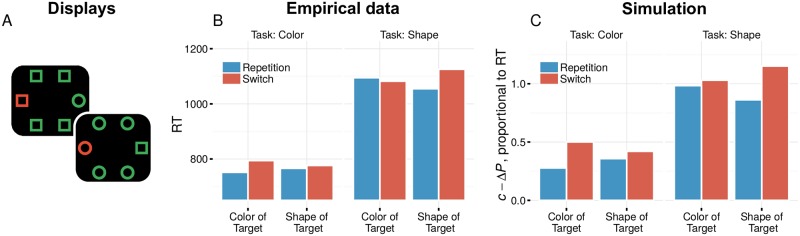
**A** Two example displays used in the simulations. Every display has two separate singleton stimuli, either a color singleton or a shape singleton. **B** Empirical data from [22]. Priming is strongly attenuated for repetitions and switches in the dimension that is irrelevant for the current task. **C** Simulations with fAIM. Bars represent the simulated priming effect Δ*P*, subtracted from a baseline *c*. A different baseline was assumed for the color task (*c* = 0.4) than for the shape task (*c* = 1.0), reflecting the apparent difference in difficulty. A similar task-dependent modulation is found, even though fAIM does not take task into account.

In this experiment, participants were presented with displays with two singletons: one shape- and one color singleton (Fig 7C). Before display onset, participants were instructed which singleton was the target, which rendered one dimension relevant and the other task-irrelevant. Priming through repetition of irrelevant target features was so small as to be statistically insignificant. However in the analysis, priming was only considered as a function of repetitions or switches of the properties of the target singleton. This ignores the effects that priming might have had on the distractor stimuli. We therefore assessed the levels of priming predicted by fAIM in this experimental design. As fAIM takes the whole image into account, it can uncover whether an absolute priming account would truly predict a different result than what was found.

Simulations were run to compute priming for all possible trial pairs in the experiment. To make model output comparable to the RTs in the empirical data, model output was transformed by subtracting the predicted change in target pop-out from a preset constant baseline (*c* − Δ*P*; with *c* = .4 for the color task and *c* = 1.0 for the shape task, to reflect their difference in overall difficulty). Subsequently, scores were aggregated for trial combinations in which the task relevant or irrelevant feature was repeated or not, separately for color- and shape tasks. Our simulation results are depicted in Fig 7, alongside the mean RTs observed in the study. Much like the empirical data, repetition effects are strongly attenuated for target repetitions and switches in the task-irrelevant dimension.

How can fAIM produce goal-dependent priming effects if it has no representation of its goal? The answer can be found in the intertrial contingencies embedded in the experiment. Consider, for example, the scenario where trial *n* is a ‘color-trial’:

- If the target on trial *n* − 1 was also defined by its color, then features that helped dissociate this target based on its color were boosted. On trial *n*, the target is again defined by color. Therefore, repetitions or switches of the *irrelevant* dimension—the shape—can either be accompanied by a repetition or by a switch in the relevant dimension. The net result then is that shape repetitions or switches produce little to no priming.
- If a target on trial *n* − 1 was defined by its shape, then features that helped dissociate this target based on its shape will be boosted. Since on trial *n* the target will be defined by color this implies that, by task design, all but one of the non-targets have the same shape as the target. The feature modulations that affect the salience of the target, will therefore similarly affect the salience of these non-targets. The net-result is that the level of pop-out of the target stimulus with respect to the rest of the display is hardly affected by both repetitions and switches of shapes. On trial *n*, the target is now defined by color. If the target’s shape repeats, the homogeneous non-targets necessarily will have the same shape as the target, and priming will yield little benefit. Similarly, if the shape switches, this would negatively affect the salience of both the target and the non-targets, and the priming effects would be limited.

A similar argument can be made for the opposite scenario, where trial *n* is a ‘shape trial’.

The simulations with fAIM thus reveal that neither a representation of goal, nor goal-dependent modulations of priming are necessary to give rise to these findings. We therefore conclude that the task-dependent modulation of priming of pop-out that was taken as evidence for goal-dependent priming, is likely to reflect an epiphenomenon caused by intertrial contingencies in the experiment, rather than a true modulation of priming by the current goals of the observer.

## Discussion

In this study we have presented fAIM, a computational model of intertrial priming in bottom-up driven visual search. The model shows how visual search trials give rise to modulations of the gains of different visual features, which causes the visual system to tune to the task at hand. fAIM was constructed with minimal assumptions and designed by implementing a priming mechanism within an established model of bottom-up visual salience. The model implements priming as a result of feature weighting, which has often been proposed to underlie priming [5, 14, 16, 50–54], sometimes in the form of dimension weighting [55]. What makes fAIM different is that it offers an in-depth perspective of what feature weighting entails for bottom-up visual processing. Our simulations with fAIM led to the following results:

- Priming of Pop-out can be successfully simulated with fAIM. It is found for classic stimulus properties such as colors, but also for less obvious ones such as ‘spikiness’ of star-shapes.
- Priming in fAIM is not restricted to a single feature that is boosted or inhibited, but results from patterns of change over all features.
- Thus, priming is part of a general adaptation to the task as a whole, and not just to target- and distractor features in isolation.
- Relational priming effects can emerge through the absolute weighting of independent features, without a representation of stimulus relation.
- Results suggestive of goal-dependent priming can emerge through the stimulus interactions within a display, without any representation of the current goal.

Much like most empirical work on priming, other computational models of priming [54, 56, 57] only consider whether target- and distractor features repeat or switch roles across trials [56, 57]. fAIM, on the other hand, models how visual experience impacts the entire visual representation, and how this in turn results in priming. Consequentially, repetition effects are not limited to the precise stimulus values that caused it, which explains how the model naturally produces relational priming and goal-dependent priming. These successes simultaneously provide new evidence that a visual representation as proposed by AIM is a reasonable model of visual processing.

Interestingly, one way to interpret the workings of fAIM is that the multivariate representation of salience inherently gives rise to a ‘relational code’. That is, those feature channels that respond strongly to, e.g., orange-red stimulus contrast are similarly involved in yellow-orange contrast, and are different from those involved in orange-yellow contrast. As such, the model demonstrates how relational guidance can *emerge* from absolute feature weighting, which is a mechanism that previous treatments of relational guidance of attention [19–21, 44, 47] never have put forward.

While the idea of feature weighting is widespread in the priming literature, it remains unclear what mechanisms would cause up- and down-regulation of feature gains. Although our simulations are consistent with various possible mechanisms, we have suggested that two processes underlie gain modulation in singleton search: accommodation to visual input, and reward-based learning through the intrinsic reward of finding a target. The idea that the up- and down-weighting of features result from dissociable processes has received some empirical support [12, 15, 58], and both processes have been alluded to in the literature. Priming has previously been linked to Repetition Attenuation, a decrease in neural activity in response to stimulus repetition, which has been observed throughout the brain in neural imaging studies [41, 42]. Many computational models of visual salience already incorporate the idea that repetition of features in time reduces their salience [25, 29, 59]. Likewise, the idea that target boosting is caused by the intrinsic reward of finding a target is supported by studies that show increased priming for targets associated with reward [60–62]. A major advantage of expressing feature modulation in terms of accommodation to input and reward-mediated boosting is that it allows us to approach priming as a natural consequence of known processes, rather than a separate mechanism introduced to explain priming effects.

In the present work we have largely focused on experiments where the target is a singleton. The simulations with fAIM show how well the results in these tasks can be captured without an explicitly represented goal or a representation of a target template; terms typically associated with ‘top-down’ attention. The presented mechanisms may not suffice to model behavior in search where the target is not at all salient, such as in conjunction search, or tasks where a distractor singleton is much more salient than the target and requires active suppression. In such cases, fAIM would still be useful as a model of the bottom-up representation that the top-down processes operate on, for example to infer search conditions that require more or less cognitive control to overcome bottom-up distraction. Also, the model could be used to gain a better understanding of what such cognitive control would actually entail; what features can and should actually be modulated to successfully select target items [23, 63]?

Finally, we feel fAIM could help dissociate the processes that determine repetition effects in feedforward-driven search and repetition effects in more effortful, goal-driven search. Strong differences do seem to exist between the two, in particular with respect to their time course. In our current implementation, the effects of priming only last a few trials, as each gain modulation overwrites previous modulations. Although this is often observed in singleton search [16, 56], priming effects in other types of search might be more persistent. We have previously reported on long-term priming in conjunction search [7] that caused persistent attentional biases towards targets that were presented more often. No such pattern was found for singleton search. The absolute feature guidance experiment that we simulated in this study can be interpreted in similar vein: priming was driven by relative stimulus values throughout a singleton search block with a constant target. However, a block with an absolute feature search had much more persistent effects, that modulated visual salience in future blocks. This is comparable to findings on the ‘attentional set’, that is acquired after training on a visual task [6, 64, 65]. Using fAIM as a basis, future work could look at the top-down modulations that these tasks require, and relate those to the longer lasting learning that occurs in such tasks.

There are also limitations to fAIM. First, the output of the model is a salience map, and how this output translates to measures of search performance (RT, eye movements or accuracy) must be further developed. Other models, have simulated the quantitative relation of priming to RT and accuracy measures in much more detail [54], and future work could combine these models to go beyond qualitative analyses of fAIM. Second, our work does not cover response- and position priming that usually co-occurs with feature priming in singleton search. However, this does not invalidate the present results as these different forms of priming have usually been found to be independent [53, 66–68]. Third, fAIM constructs its salience map at a single spatial scale, ignoring the hierarchical organization of the visual pathway. One can imagine a hierarchical visual representation based on similar principles, where higher order maps encode regularities in the low-level output [38, 59]. This might allow for interactions between stimuli across larger spatial distances that are currently neglected in fAIM.

We conclude that fAIM offers new insight into visual priming by emphasizing the wealth of information in the visual representation. The model describes how this wealth can be used by the brain to adapt our senses to the stimuli it will likely have to deal with in the future. This highlights how priming is more than just a peculiarity arising in visual search experiments. Instead, it is an expression of an adapting brain in a dynamic environment.

## Supporting information

S1 Text Model implementation details. This supplementary document gives an overview of the computations in the AIM saliency model, and its extension to fAIM as applied in the simulations.
(PDF)

S2 Text Simulation details. This supplementary document describes the implementation details of the simulations. In particular, it describes how the stimuli and displays were defined.
(PDF)
