# Supplementary material for: You prime what you code: The fAIM model of priming of pop-out
Source: PLoS One. 2017 Nov 22;12(11):e0187556. doi: 10.1371/journal.pone.0187556 (PMC5699828; doi:10.1371/journal.pone.0187556)
Supplement: S1 Text — This supplementary document gives an overview of the computations in the AIM saliency model, and its extension to fAIM as applied in the simulations. (PDF) [file pone.0187556.s001.pdf]

Supporting Information

S1 Text

Implementation details of the model: The main text only gives a description of AIM and fAIM that is largely conceptual, and various technical details were omitted. These details are listed in the following paragraphs. Note that the basis of our model, the implementation of AIM, was effectively a reimplementation of the code for AIM available from the homepage of N. B. Bruce (`http://www-sop.inria.fr/members/Neil.Bruce/`).

Visual Features The visual features were derived from 40,000 image patches of 31 × 31 × RGB generated by extracting 5 patches from 8,000 images. The images were drawn from the SUN database [3], but we excluded images that were black and white, were less than 300 px wide or tall, or that were labeled ''outliers' in the dataset.

Features were computed by running JADE independent component analysis [4] (ICA), after the data had been whitened by a principal component analysis that left only those components to account for at least 95% of the variance in the data. Different runs with newly sampled image patches resulted in highly corresponding features, though the number of features varied around 54±3. In the simulations used here, a single feature space of 54 features was used.

Self-information and visual pop-out Input images were decomposed into 31×31 patches. To correct for edge artifacts, images were reflected at the edges, although the stimuli in our images were placed at sufficient distance from this boundary not to be affected. Convolution of the image with the ICA unmixing matrix yields the response of each feature at each location.

These responses across images were summarized into a histogram of 1,000 bins for each feature channel. The histogram was smoothed by convolution with a Gaussian filter, which had a σ of .05s, where s is the standard deviation computed across the bins in the histogram. The resulting bin values were then normalized to sum to 1.

The resulting bin values were used to determine the probability $p_f(x)$ of all stimulus values at every location x. These were transformed into salience values, or

self-information, via $S_f(x) = -\log(p_f(x))$.

Insight that the salience value in AIM is composed of the independent contribution of all feature channels is clear when the salience computation is rewritten as:

$$\begin{aligned} S(x) &= -\log(p(x)) \\ &= \sum_f -\log(p_f(x)) = \sum_f S_f(x) \end{aligned} \tag{1}$$

where f refers to each feature channel; x refers to any location in the image, and is omitted from the upcoming equations.

The fAIM model fAIM generalizes this model and assumes all feature channels have a dynamic weight or 'gain' g_f that determines their contribution to overall salience:

$$S = \sum_f g_f S_f = \mathbf{g} \cdot \mathbf{S} \tag{2}$$

The average salience level for each stimulus $\bar{S}$ is computed, which is used to compute stimulus pop-out (P) as $\bar{S}^T - \bar{S}^A$. Here, T implies that the Target salience is used, but pop-out can be computed for each distractor stimulus in the same manner.

As explained in the main text, the same computation *within* feature channels determines the intertrial gain modulation for each feature (Δg_f) via:

$$\Delta g_f = w_e \bar{S}_f^T - w_i \bar{S}_f^A \tag{3}$$

with $w_i = w_e = 1$ for all simulations. The gain vector used on a subsequent trial resulted from adding $\mathbf{\Delta g}$ and bounding gain values via a sigmoid function:

$$\begin{aligned} \mathbf{g}_{n+1} &= \sigma(\mathbf{g}_n + \Delta \mathbf{g}_n) \\ \text{with } \sigma(g) &= \frac{u}{1 + e^{-k(g-m)}} \end{aligned} \tag{4}$$

The gains were bounded between 0 and $u =^2 /_x$ with middle point $m =^1 /_x$, where $x = 54$ was the number of feature channels in the visual representation. All gains were initialized at m, so channel gains summed to 1. The slope parameter $k = 100$ was chosen so that in the present values, the sigmoid would only affect values after repeated

modulations in the same direction, as can be observed in Fig. 3B.

Our model offers a mechanistic, qualitative explanation of a wide range of priming effects. For the most part, varying the model parameters merely scale the found effects, which does not lead to different conclusions than those drawn in the present work. However, future work could explore the quantitative relation between salience and search performance in more detail, and in that case these parameters could be varied to capture variability across paradigms or individuals. For example, the w_e/w_i ratio could be changed to capture dissociable effects of distractor inhibition and target facilitation in priming [1], and the slope parameter k might be modulated to capture variability in the time course of priming effects [2].

References

1. Lamy D, Antebi C, Aviani N, Carmel T. Priming of Pop-out provides reliable measures of target activation and distractor inhibition in selective attention. Vision Research. 2008;48(1):30–41.

2. Martini P. System identification in Priming of Pop-Out. Vision Research. 2010;50(21):2110–2115.

3. Xiao J, Hays J, Ehinger KA, Oliva A, Torralba A. SUN database: Large-scale scene recognition from abbey to zoo. In: 2010 IEEE Conference on Computer Vision and Pattern Recognition (CVPR); 2010. p. 3485–3492.

4. Cardoso JF. High-Order Contrasts for Independent Component Analysis. Neural Computation. 1999;11(1):157–192.
