# Supplementary material for: You prime what you code: The fAIM model of priming of pop-out
Source: PLoS One. 2017 Nov 22;12(11):e0187556. doi: 10.1371/journal.pone.0187556 (PMC5699828; doi:10.1371/journal.pone.0187556)
Supplement: S2 Text — This supplementary document describes the implementation details of the simulations. In particular, it describes how the stimuli and displays were defined. (PDF) [file pone.0187556.s002.pdf]

Supporting Information

S2 Text

Details of simulated stimulus displays All stimuli were drawn on an isolated 'stimulus patch', a 301×301 grid. This grid was then resized to 76×76 via bilinear interpolation to reduce aliasing artifacts of drawing. These patches were then assembled appropriately to construct stimulus displays. The following paragraphs describe how patches were constructed for each stimulus type.

Stimuli Color and luminance stimuli were filled circles, with a diameter spanning the full patch width. Colors were generated from color maps integrated in the `matplotlib` plotting library [3]. In the initial red-green color priming simulation (Fig. 2), the two colors at 10% and 90% of the `RdYlGn` map were drawn. For the relational priming simulations, the `YlOrRd` color map was used for the Yellow-Red dimension (Fig. 4B), and the `BuGn` map Blue-Green stimuli (Fig. 4C). Colors were drawn as four evenly spaced points on this map from 20% – 80%. The absolute- and relative capture simulation (Fig. 6A) used five colors from the `YlOrRd` color map, drawn from the same range. For all these simulations, stimuli were rendered isoluminant to the model by normalizing the RGB color vectors.

For simulations of relational priming in size and luminance, values corresponded to those reported for their associated experiments [1]. The luminance stimulus set consisted of four gray circles with 35%–80% intensity (Fig. 4D). Size stimuli were white circles, with diameters at 30% - 90% of the patch width (Fig. 4E).

Star-stimuli were defined by connecting p points on a circle in such a way that every point i is connected to the next point $i + q$ (wrapping around the circle when applicable). Different regular shapes can be generated by choosing different values for p and q In these simulations, we used $p : q = \{5 : 2,\ 7 : 3,\ 9 : 4,\ 11 : 5\}$ which produces the 5-, 7,- 9- & 11-point star shapes depicted in Fig. 4F. Polygon shapes (Fig. 4G) were constructed in a similar way but with $q = 1$, i.e. by connecting adjacent points on the circle. For the triangle, a circle was used with a diameter that spanned the full patch width. Higher order polygons were scaled down so their surface area matched that of the triangle.

In goal-dependent priming, stimuli were either red or green, defined like before. In the original experiment [2], stimuli were landolt-C's and landolt-squares. We mimicked that appearance by drawing filled circles and squares, then leaving the center 60% of the surface blank.

Display configurations The 'basic displays' that were used in most simulations were 4 stimulus patches placed in a grid in the center of a 376×376 image on a black background. The spacing between them was 76px, identical to the patch size.

The simulations of the relative-shape priming experiments used displays of 376×413, with seven stimuli placed equidistantly on a circle with a 152 px radius. The simulations to study goal-dependent priming used similar displays but contained only six display items.

The simulations of absolute and relative attentional capture used singleton displays of 376×466 px, with the displays identical to the grids used in the basic displays but with an additional stimulus on the right at the same horizontal distance. The feature search displays had an identical layout as the shape displays described above.

References

1. Becker SI. The role of target-distractor relationships in guiding attention and the eyes in visual search. Journal of Experimental Psychology: General. 2010;139(2):247.

2. Fecteau JH. Priming of pop-out depends upon the current goals of observers. Journal of Vision. 2007;7(6):1.

3. Hunter JD. Matplotlib: A 2D Graphics Environment. Computing in Science & Engineering. 2007;9(3):90–95.
